# Cost-Effectiveness of Single- Versus Generic Multiple-Tablet Regimens for Treatment of HIV-1 Infection in the United States

**DOI:** 10.1371/journal.pone.0147821

**Published:** 2016-01-25

**Authors:** Donna E. Sweet, Frederick L. Altice, Calvin J. Cohen, Björn Vandewalle

**Affiliations:** 1 Internal Medicine, The University of Kansas School of Medicine–Wichita, Wichita, Kansas, United States of America; 2 Section of Infectious Diseases, Yale University School of Medicine, New Haven, Connecticut, United States of America; 3 CRI New England, Boston, Massachusetts, United States of America; 4 Exigo Consultores, Lisbon, Portugal; British Columbia Centre for Excellence in HIV/AIDS, CANADA

## Abstract

**Background:**

The possibility of incorporating generics into combination antiretroviral therapy and breaking apart once-daily single-tablet regimens (STRs), may result in less efficacious medications and/or more complex regimens with the expectation of marked monetary savings. A modeling approach that assesses the merits of such policies in terms of lifelong costs and health outcomes using adherence and effectiveness data from real-world U.S. settings.

**Methods:**

A comprehensive computer-based microsimulation model was developed to assess the lifetime health (life expectancy and quality adjusted life-years—QALYs) and economic outcomes in HIV-1 infected patients initiating STRs compared with multiple-table regimens including generic medications where possible (gMTRs). The STRs considered included tenofovir disoproxil fumarate/emtricitabine and efavirenz or rilpivirine or elvitegravir/cobicistat. gMTRs substitutions included each counterpart to STRs, including generic lamivudine for emtricitabine and generic versus branded efavirenz.

**Results:**

Life expectancy is estimated to be 1.301 years higher (discounted 0.619 QALY gain) in HIV-1 patients initiating a single-tablet regimen in comparison to a generic-based multiple-table regimen. STRs were associated with an average increment of $26,547.43 per patient in medication and $1,824.09 in other medical costs due to longer survival which were partially offset by higher inpatients costs ($12,035.61) with gMTRs treatment. Overall, STRs presented incremental lifetime costs of $16,335.91 compared with gMTRs, resulting in an incremental cost-effectiveness ratio of $26,383.82 per QALY gained.

**Conclusions:**

STRs continue to represent good value for money under contemporary cost-effectiveness thresholds despite substantial price reductions of generic medications in the U. S.

## Introduction

Innovations in antiretroviral therapy (ART) have dramatically altered the natural history of HIV infection, transforming it into a manageable chronic disease [1]. ART has markedly increased survival in people living with HIV (PLH) and extended life expectancy such that there are little differences between those with and without HIV [2]. Nonetheless, PLH still have a slightly shorter lifespan relative to the general population with important challenges remaining [3]. Delayed access to treatment and suboptimal ART adherence crucially influence poor outcomes, including the increased risk of hospitalization and death [4].

Increasing life expectancy contribute to increased total lifetime costs of HIV care, posing challenges for funders despite annual costs of treating someone with HIV having remained fairly constant over the years [5]. Many different strategies have been proposed to reduce the economic burden of HIV [6]. One strategy is the incorporation of generic medications into ART regimens as soon as they become available. That has led to suggestions of potentially including less efficacious drugs as well as more complex regimens under the expectation of monetary savings [7].

At first glance, these strategies seem appealing because they appear to present significant economic savings. Caution is warranted in interpreting these results, however, because some of these economic analyses are focused mainly on ART costs, ignoring other important costs such as inpatient and outpatient costs, or even wider economic societal implications, that may account for up to 45% of the total lifetime costs of HIV care [8–10]. Moreover, there are compelling data from a meta-analysis that suggest that treating with recommended treatments, rather than alternative recommendations is associated with markedly improved outcomes [11].

There is evidence that antiretroviral pill burden and treatment adherence are key determinants of the risk of hospitalization and inpatients costs [4, 12]. Patients on a once-daily single-tablet regimen (STR) have been shown to be significantly more likely to be highly adherent (≥95%) to therapy than patients who received a multiple-tablet regimen (MTR), and that improved adherence among patients treated with STR is associated with a lower risk of hospitalization and reduce healthcare costs, including significantly lower inpatient costs [12]. These important factors are crucial for cost-effectiveness studies and should be incorporated into models to better inform efficient resource allocation.

Also of concern is the justification of monetary savings being considered at the expense of potential health losses without adequately addressing the fact that societal value for a health loss is regarded to be substantially higher than the societal value for a health gain, i.e. monetary saving required to move from optimal to sub-optimal therapy are higher than the maximum willingness to pay for a health gain obtained by moving from lower to a higher health status [13].

The present study includes a computer-based microsimulation model of HIV disease progression based on adherence and effectiveness data from real-world settings in the US to comprehensively assess the long-term health and economic outcomes of HIV infected patients initiating single-tablet regimens (STRs) compared with multiple-tablet regimens including generic medications when possible (gMTRs).

## Materials and Methods

### Overview

In a simulation study two treatment strategies were considered for HIV-1 infected adult patients initiating first-line ART (Table 1): a branded 1-pill strategy (STR), and a generic-based 3-pill strategy (gMTR). Evaluated STRs were based on a backbone of tenofovir and emtricitabine (TDF/FTC), whereas gMTRs were based on a backbone of tenofovir and generic lamivudine (TDF+g3TC). Third agents and the percentage of patients initiating them did not depend on the treatment strategy chosen, and consisted of efavirenz (branded for STR: EFV; generic for MTR: gEFV), rilpivirine (RPV) and cobicistat-boosted elvitegravir (EVG/COBI).

**Table 1:** First-line antiretroviral therapy strategies, regimen components and market shares.

STR Strategy	gMTR Strategy	% Patients Initiating
EFV/TDF/FTC	gEFV+TDF+g3TC	30.4%^a^ [14]
RPV/TDF/FTC	RPV+TDF+g3TC	26.1%^a^ [14]
EVG/COBI/TDF/FTC	EVG/COBI+TDF+g3TC	43.5%^a^ [14]

COBI, cobicistat; EFV, efavirenz; EVG, elvitegravir; FTC, emtricitabine; g3TC, generic lamivudine; gEFV, generic efavirenz; gMTR, generic multiple-tablet regimen; RPV, rilpivirine; STR, single-tablet regimen; TDF, tenofovir.

^a^Percentage of patients initiating corresponding ART regimen.

The expected long-term, comparative economic and clinical impact of the proposed treatment strategies were analyzed using a microsimulation cost-effectiveness model for HIV treatment. Given the chronic and progressive nature of the disease, the model considered a lifetime time horizon. The economic impact was measured in terms of the expected total per-person cost to the U.S. health system, whereas the clinical impact or effectiveness was measured in terms of corresponding expected quality-adjusted life-years (QALY). Following U.S. recommendations, an annual 3% discount rate was applied to both costs and effectiveness. Deterministic sensitivity analysis was used to assess parameter uncertainty, where possible.

### Microsimulation model

**Fig 1:**
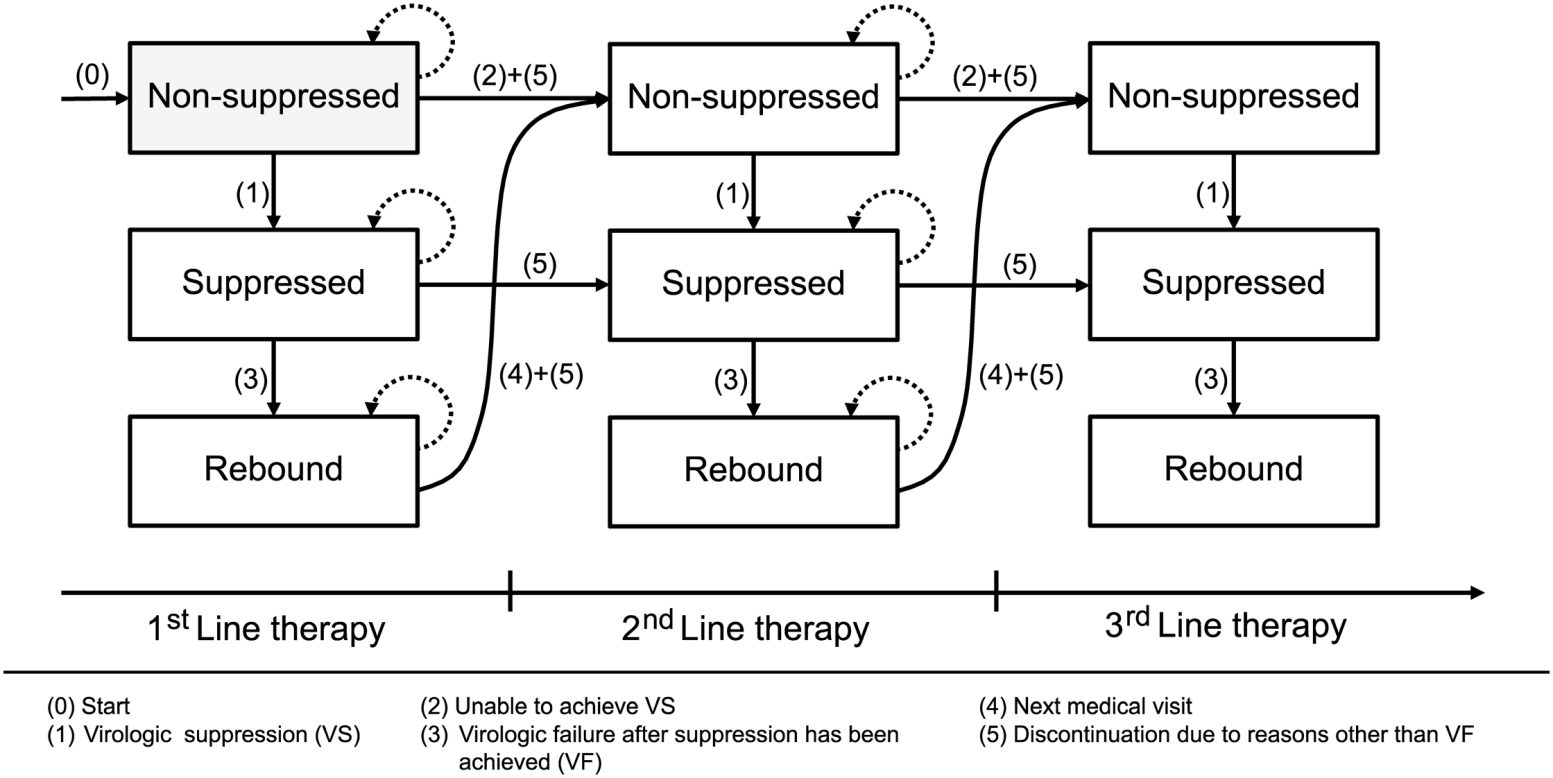
State-transition model at the core of the microsimulation model, consisting of 3 main health-states (‘non-suppressed’, ‘suppressed’ and ‘rebound’) within each subsequent therapy line. Arrows indicate the possible transitions between states and therapy lines.

At the core of the microsimulation model lies a state-transition model (Fig 1), in which patients’ virologic response to ART is simulated, in monthly updates, to belong to one of 3 mutually exclusive health states: “non-suppressed”, “suppressed” and “rebound”.

Patients enter the model in the “non-suppressed” state (transition 0), where they initiate first-line therapy. Within this state, a monthly probability of virologic suppression (VS), applied during the first 6 months of therapy, determines if and when a patient moves to the “suppressed” state, and can be considered a responder to therapy (transition 1). Patients in the “non-suppressed” state that do not achieve VS within the first 6 months of therapy move to the “non-suppressed” state of the subsequent therapy line at the next scheduled medical visit (transition 2).

Once transitioned to the “suppressed” state, from the 7th month of therapy onwards, patients are subject to a monthly probability of virologic failure after suppression has been achieved (VF). Upon VF, patients move to the “rebound” state (transition 3), where they stay until the next scheduled medical visit, upon which they move to the “non-suppressed” state of the subsequent therapy line (transition 4).

Irrespective of their state of virologic suppression, patients are continuously subject to a monthly probability of discontinuation due to reasons other than VF (transitions 5). Upon discontinuation while in the “suppressed” state, patients immediately transition to the “suppressed” state of the subsequent therapy line. For the remaining non-virologically suppressed states, patients transition to the “non-suppressed” state of the subsequent therapy line. Finally, patients are also continuously subject to a monthly probability of death (not shown in Fig 1).

The same rules as above, conditional on the health states patients are in and the time they have spent in them, are applied to each subsequent therapy line. Six active therapy lines are modelled. Upon failing or discontinuing sixth-line therapy, patients are assumed to move to a non-suppressed state, where they remain until death. Throughout the simulation, medical visits are scheduled every 3 months.

**Fig 2:**
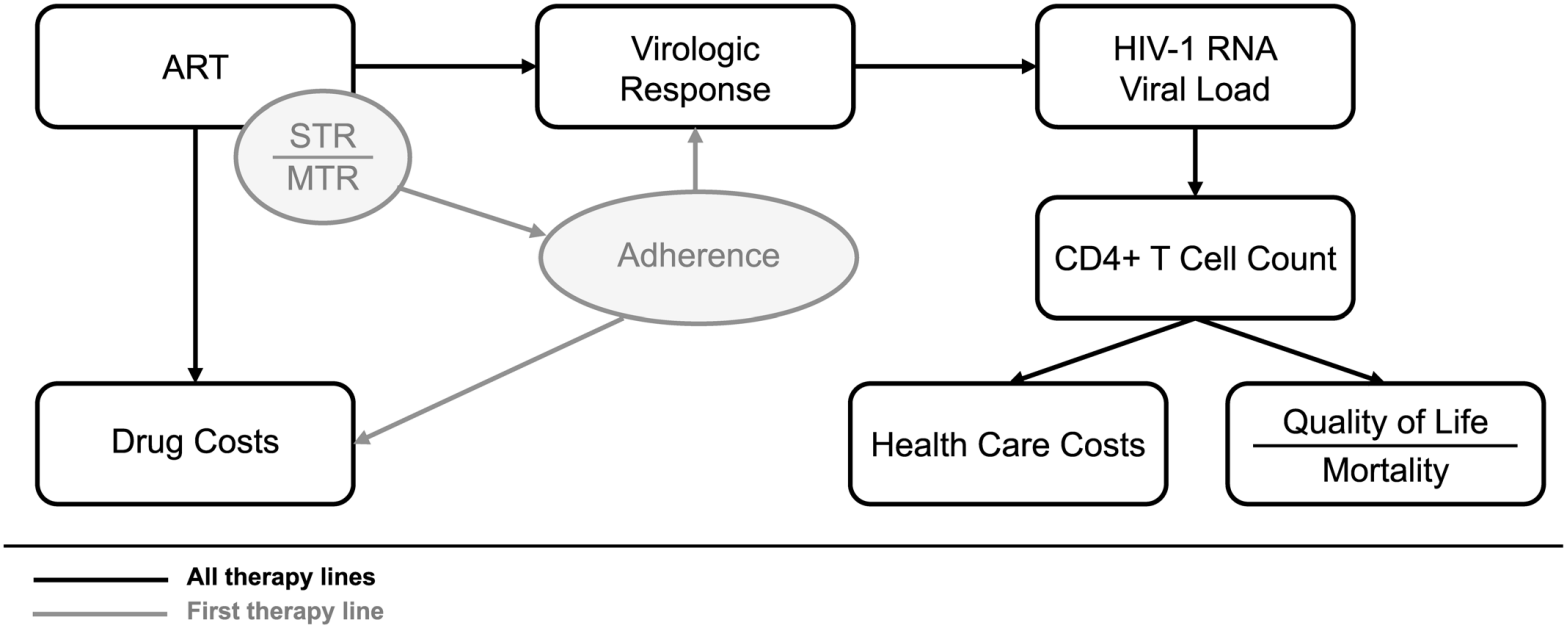
Model influence diagram. Within each therapy line, the choice of ART regimen determines virologic response and drug costs. Virologic response, in turn, influences the evolution of HIV-1 RNA viral load and CD4+ T cell counts over time. Health care costs and QALY, through QoL and mortality, are dependent on CD4+ T cell counts. For first-line therapy, differential adherence between STR and MTR further influences virologic response and drug costs.

In each model run, a cohort of patients is simulated with key baseline characteristics (i.e. age, gender, HIV-1 RNA viral load and CD4+ T cell count) and first-line treatment strategy (STR vs. gMTR) dependent adherence levels. Every patient is simulated under both an STR and a gMTR first-line strategy (with equivalent third agent). Differential adherence between STRs and gMTRs is assumed to influence virologic response for first-line therapy only. Subsequent therapy lines are the same for all patients, irrespective of first-line STR or gMTR strategy. At each monthly cycle, the characteristics associated with each patient, including age, HIV-1 RNA viral load, CD4+ T cell count, quality of life (QoL), and health care and drug costs, are tracked and updated. An influence diagram relating the different model components can be found in Fig 2. Details are given below.

### Model Parameterization

#### Baseline characteristics

With the aim of modeling a general ART-naïve HIV infected population, correlated baseline HIV-1 RNA viral load and CD4+ T cell count distributions were calibrated with data from Rodriguez et al. (2006) [15], presenting a mean viral load of 4.01 log10 HIV-1 RNA cps/mL and a mean CD4+ T cell count of 525 cells/mm^3^ (Table 2). Mean age at ART initiation was assumed to be 43 years, with 84% of the population being male (Table 2) [7].

**Table 2:** Baseline model characteristics.

Variable	Value	Reference
Age—Mean (SD), years	43 (12)	[7]
Gender—Male, %	84	[7]
Viral load × CD4 count—Mean (correlation matrix), log_10_ RNA cps/mL × cells/mm^3^	$\left[\begin{array}{c} 4.01\\ 525 \end{array}\right]\;\left(\left[\begin{array}{cc} 1.1 & -0.37\\ -0.37 & 228 \end{array}\right]\right)$	[15]

SD, standard deviation.

**Fig 3:**
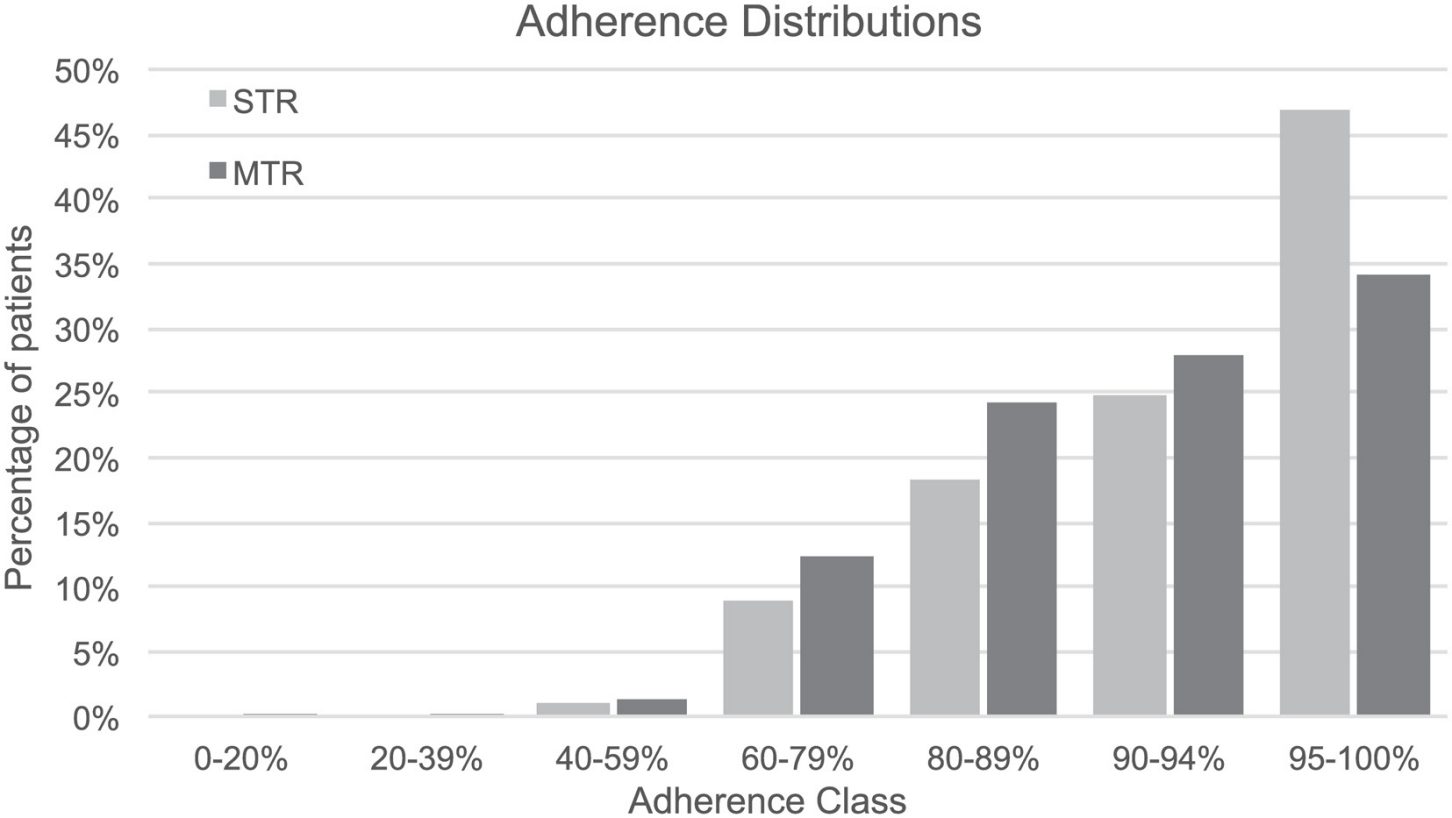
Distribution of real-world average adherence levels, stratified by first-line treatment strategy. MTR, multiple-tablet regimen; STR, single-tablet regimen.

The proportions of patients receiving either of the three regimens considered in each of the treatment strategies (Table 1) were based on US market shares [14]. For patients receiving RPV-based ART, baseline viral load was simulated below 5 log10 RNA cps/mL, to match the corresponding label [16]. Real-world adherence levels to first-line therapy were based on Sweet et al. (2014) [17] and Sax et al. (2012) [4] and are presented in Fig 3.

#### State-transition probabilities

The monthly probabilities of virologic suppression and virologic failure for all first and subsequent therapy lines considered (Table 3), were calibrated on the basis of an external cohort state-transition model (corresponding to a single therapy line from Fig 1) as for the time-dependent percentage of patients in the “suppressed” state to approximate the time-dependent percentage of patients with HIV-1 RNA viral load below 50 cps/mL observed in the selected clinical trials. The treatment sequence from 2nd to 6th therapy line was based on expert opinion and efficacy derived from the corresponding trials.

**Table 3:** Calibrated monthly virologic suppression and virologic failure probabilities.

Therapy Line	ART Regimen	Monthly Probability of Virologic		Source
		Suppression	Failure	
**1**^**st**^ **Line**	**EFV/TDF/FTC**	42.38%	0.03%	GS-236-102 [18–20]
**STR**	**RPV/TDF/FTC**	56.92%	0.34%	STAR Study [21, 22]
	**EVG/COBI/TDF/FTC**	44.63%	0.03%	GS-236-102 [18–20]
**1**^**st**^ **Line**	**gEFV+TDF+g3TC**	33.27%	0.16%	Study 903 [23, 24]
**gMTR**	**RPV+TDF+g3TC**	47.25%	1.69%	Assumption^a^
	**EVG+COBI+TDF+g3TC**	35.33%	0.16%	Assumption^a^
**2**^**nd**^ **Line**	**ATV/r + TDF/FTC**	9.12%	0.82%	BMS Study 045 [25, 26]
**3**^**rd**^ **Line**	**DRV/r + TDF/FTC**	12.46%	0.33%	POWER 1–2 [27–31]
**4**^**th**^ **Line**	**DTG + TDF/FTC**	28.70%	2.09%	SAILING [32, 33]
**5**^**th**^ **Line**	**DRV/r + DTG + TDF/FTC**	21.14%	1.65%	VIKING [34]
**6**^**th**^ **Line**	**ENF + OBR**^b^	43.69%	1.13%	TORO 1–2 [35, 36]

/r, ritonavir boosted; ATV, atazanavir; COBI, cobicistat; DRV, darunavir; DTG, dolutegravir; EFV, efavirenz; ENF, enfuvirtide; EVG, elvitegravir; FTC, emtricitabine; g3TC, generic lamivudine; gEFV, generic efavirenz; OBR, optimized background regimen; RPV, rilpivirine; TDF, tenofovir.

^a^Determined by applying the odds ratio between the corresponding probabilities of the gEFV+TDF+g3TC and EFV/TDF/FTC regimens to the probabilities of the RPV/TDF/FTC and EVG/COBI/TDF/FTC regimens.

^b^Calibrated on the percentage of patients remaining on treatment, assuming that for a multi-drug resistant end-of-line population, reaching a viral load below 50 RNA cps/mL is not the criteria on which viral failure is determined [35].

Simplified selection criteria adopted for the incorporated clinical trials were: 1) phase II or III randomized clinical trials, with preference to phase III studies; 2) patient characteristics in terms of the number of previous therapy lines that best fitted the therapy line in question; 3) follow-up data in at least two points in time, with preference for the most recent studies with more available time-points and longer follow-up (e.g.: efficacy and safety results at 48, 96 and 144 weeks).

First-line gMTR, EFV+TDF+3TC has been studied in clinical trial setting in Study 903 [23, 24] and results from this trial, as in other publish cost-effectiveness analysis [7], were assumed to reflect those of gEFV+TDF+g3TC. RPV+TDF+g3TC and EVG/COBI+TDF+g3TC, have not been studied in a clinical trial setting. To overcome this lack of information, viral suppression and failure probabilities for these regimens were determined by applying the odds ratio between the corresponding probabilities of the gEFV+TDF+g3TC and EFV/TDF/FTC regimens to the probabilities of the RPV/TDF/FTC and EVG/COBI/TDF/FTC regimens.

**Fig 4:**
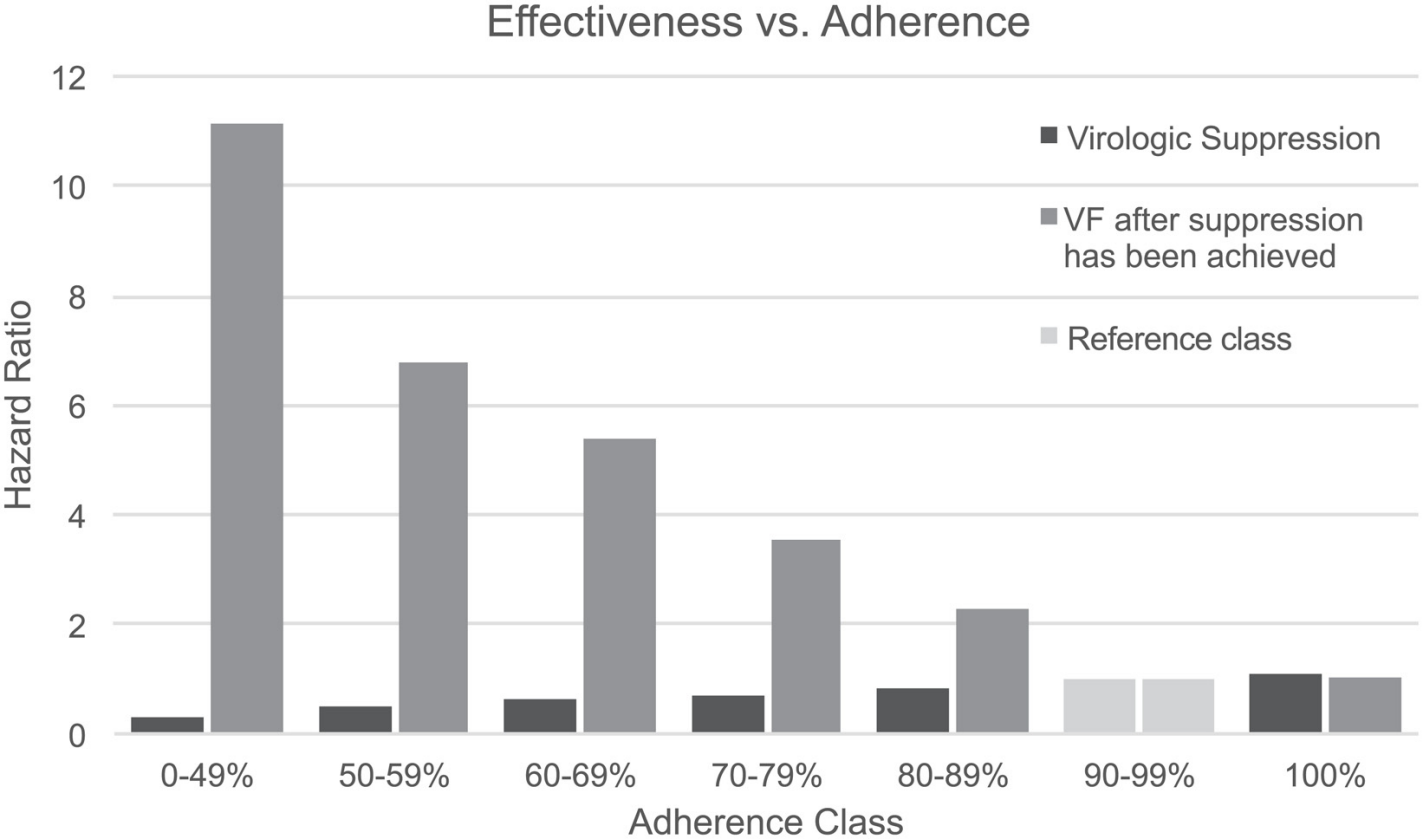
Hazard ratios of virologic suppression and virologic failure for different adherence classes. VF, virological failure.

The first-line therapy virologic suppression and failure probabilities presented in Table 3 were considered for high levels of average adherence usually observed in clinical trial settings. To accommodate for declining levels of virologic response typically associated with real-life lower adherence levels (Fig 3), monthly suppression and failure probabilities were adjusted using hazard ratios adapted from Nachega et al. (2007) [37] (Fig 4), using the 90–99% adherence class as a reference class for the base-case values in Table 3.

Monthly probabilities for discontinuation due to other reasons were determined from the clinical trials used for the calibration of virologic response probabilities, considering discontinuations for all reasons (including adverse events) except virologic failure (lack of efficacy) and death (Table 4).

**Table 4:** Calibrated monthly virologic suppression and virologic failure probabilities.

Therapy Line	ART Regimen	Monthly Probability of Discontinuation		Source
		First year	Subsequent Years	
**1**^**st**^ **Line**	**EFV/TDF/FTC**	1.09%	0.50%	GS-236-102 [18–20]
**STR**	**RPV/TDF/FTC**	0.91%	0.61%	STAR Study [21, 22]
	**EVG/COBI/TDF/FTC**	0.84%	0.56%	GS-236-102 [18–20]
**1**^**st**^ **Line**	**gEFV+TDF+g3TC**	1.36%	0.46%	Study 903 [23, 24]
**gMTR**	**RPV+TDF+g3TC**	1.14%	0.56%	Assumption^a^
	**EVG+COBI+TDF+g3TC**	1.05%	0.52%	Assumption^a^
**2**^**nd**^ **Line**	**ATV/r + TDF/FTC**	0.71%	0.79%	BMS Study 045 [25, 26]
**3**^**rd**^ **Line**	**DRV/r + TDF/FTC**	1.04%	1.28%	POWER 1–2 [27–31]
**4**^**th**^ **Line**	**DTG + TDF/FTC**	0.94%	0.10%	SAILING [32, 33]
**5**^**th**^ **Line**	**DRV/r + DTG + TDF/FTC**	1.15%	1.22%	VIKING [34]
**6**^**th**^ **Line**	**ENF + OBR**	1.83%	2.13%	TORO 1–2 [35, 36]

/r, ritonavir boosted; ATV, atazanavir; COBI, cobicistat; DRV, darunavir; DTG, dolutegravir; EFV, efavirenz; ENF, enfuvirtide; EVG, elvitegravir; FTC, emtricitabine; g3TC, generic lamivudine; gEFV, generic efavirenz; OBR, optimized background regimen; RPV, rilpivirine; TDF, tenofovir.

^a^Similar procedure as for the assumptions in Table 3.

Monthly probabilities of death were determined by multiplying CD4+ T cell count dependent standardized mortality ratios (SMR) to gender and age specific base mortality rates reported by the Center for Disease Control and Prevention (CDC) national vital statistics (Table 5) [38].

**Table 5:** Standardized mortality ratios as a function of CD4+ T cell count.

CD4+ T cell count (cells/mm^3^)	Standardized mortality ratio
≥500	2.5
350–499	3.5
200–349	5.6
≤199	30.3

#### Evolution of HIV-1 RNA viral load and CD4+ T cell count

While in the “suppressed” state (Fig 1), patients have their viral load set, and maintained, at a constant low level (below 50 cps/mL). Meanwhile, CD4+ T cell counts increase over time in a logarithmic fashion. The monthly logarithmic rate of increase for the different therapy lines and regimens considered were determined on the reported 48 week mean change from baseline in CD4+ T cell count observed in the clinical trials used for virologic response calibration (Table 6).

**Table 6:** 48 Week CD4+ T cell count increase.

Therapy Line	ART Regimen	CD4+ T cell count increase at 48 weeks	Source
**1**^**st**^ **Line**	**EFV/TDF/FTC**	206	GS-236-102 [18–20]
**STR**	**RPV/TDF/FTC**	200	STAR Study [21, 22]
	**EVG/COBI/TDF/FTC**	239	GS-236-102 [18–20]
**1**^**st**^ **Line**	**gEFV+TDF+g3TC**	205	Study 903 [23, 24]
**gMTR**	**RPV+TDF+g3TC**	199	Assumption^a^
	**EVG+COBI+TDF+g3TC**	230	Assumption^a^
**2**^**nd**^ **Line**	**ATV/r + TDF/FTC**	110	BMS Study 045 [25, 26]
**3**^**rd**^ **Line**	**DRV/r + TDF/FTC**	102	POWER 1–2 [27–31]
**4**^**th**^ **Line**	**DTG + TDF/FTC**	162	SAILING [32, 33]
**5**^**th**^ **Line**	**DRV/r + DTG + TDF/FTC**	110	VIKING [34]
**6**^**th**^ **Line**	**ENF + OBR**	119	TORO 1–2 [35, 36]

/r, ritonavir boosted; ATV, atazanavir; COBI, cobicistat; DRV, darunavir; DTG, dolutegravir; EFV, efavirenz; ENF, enfuvirtide; EVG, elvitegravir; FTC, emtricitabine; g3TC, generic lamivudine; gEFV, generic efavirenz; OBR, optimized background regimen; RPV, rilpivirine; TDF, tenofovir.

^a^Similar procedure as for the assumptions in Table 3.

For patients in the “rebound” state, viral load is reset to the highest level ever observed since treatment initiation (≥ 50 RNA cps/mL), after which it keeps increasing on a log10 scale, by an annual rate of 0.11 while CD4+ T cell counts are greater than or equal to 200 cells/mm^3^ and by an annual rate of 0.17 otherwise [39]. CD4+ T cell counts are similarly reset to the lowest level ever observed, afterwards further decreasing as a function of viral load, determined on the basis of data from the Multicenter AIDS Cohort Study [40].

For patients in the “non-suppressed” state that will never suppress, viral load and CD4+ T cell count evolution is equivalent to that of patients in the “rebound” state. For the remaining patients, viral load and CD4+ T cell count are equivalent to the evolution discussed for patients in the “suppressed” state.

#### Costs and Quality of Life

Costs accounted for in the model include ART costs, hospitalizations (inpatient) costs, and other medical expenses (cost of prophylaxis therapy for opportunistic infections, outpatient treatment, emergency department visits, non-HIV medication and laboratory testing [8]). All costs were inflated, where necessary, to 2014 USD using publically available health-care consumer price indices [41].

Unit ART costs were based on data from First Databank, (April 1^st^, 2015; Table 7). The cost of generic EFV was assumed to be 25% of its branded equivalent. The cost for the optimized background regimen (OBR) of sixth-line therapy was based on the OBR composition presented in Hill et al. (2011) [42].

**Table 7:** Annual ART costs (First Databank, April 1^st^, 2015).

Therapy Line	ART Regimen	AnnualART Cost
**1**^**st**^ **Line**	**EFV/TDF/FTC**	$25,874.00
**STR**	**RPV/TDF/FTC**	$24,975.86
	**EVG/COBI/TDF/FTC**	$29,896.54
**1**^**st**^ **Line**	**gEFV+TDF+g3TC**	$17,143.14
**gMTR**	**RPV+TDF+g3TC**	$24,167.86
	**EVG+COBI+TDF+g3TC**	$29,088.55
**2**^**nd**^ **Line**	**ATV/r + TDF/FTC**	$34,159.88
**3**^**rd**^ **Line**	**DRV/r + TDF/FTC**	$34,049.88
**4**^**th**^ **Line**	**DTG + TDF/FTC**	$31,649.88
**5**^**th**^ **Line**	**DRV/r + DTG + TDF/FTC**	$66,123.88
**6**^**th**^ **Line**	**ENF + OBR**	$59,193.30

Inpatient costs and other medical costs were calculated based on the patient’s CD4+ cell count (Table 8). Real-world evidence suggests a 45% decrease in hospitalizations for patients on STR therapy as compared to those on gMTR therapy [43], as such, it was assumed that first-line STR therapy inpatient costs were 55% of the corresponding first-line gMTR therapy costs. This value was subject to sensitivity analysis to reflect other studies evidence [4, 12].

**Table 8:** Annual inpatient and other medical costs as a function of CD4+ T cell count.

CD4+ T cell count (cells/mm^3^)	Inpatient Costs	Other Medical Costs
≤ 50	$32,018.95	$8,529.76
51–200	$12,463.18	$5,919.56
201–350	$5,660.61	$4,433.19
351–500	$3,198.01	$4,224.74
> 500	$1,386.67	$4,182.01

CD4+ T cell count dependent health-related QoL utility values for quality adjusted life year calculation were taken from Kauf et al. (2008) [44] and are presented in Table 9.

**Table 9:** Quality of life utility values as a function of CD4+ T cell count.

CD4+ T cell count (cells/mm^3^)	QoL Utility
≥ 500	0.946
350–499	0.933
200–349	0.931
100–199	0.853
< 100	0.781

## Results

The model simulated 200,000 hypothetical individuals initiating ART (100,000 for STRs and 100,000 for gMTRs) using real-world evidence. All results represent the averages of each group of hypothetical individuals (STR vs. gMTR) and are discounted at 3.0% per annum.

After incorporating real-world evidence, over the lifetime of a patient an additional discounted 0.619 QALYs (14.466 vs. 13.847) were estimated to be gained with STRs compared with gMTRs (Table 10). Single-tablet regimens were associated with an incremental $26,547.43 per patient in medication costs and $1,824.09 in other medical costs due to longer survival in comparison to gMTRs (Table 10). STRs, however, were estimated to reduce inpatient costs by $12,035.61 relative to gMTRs (Table 10). Overall, STRs were associated with incremental lifetime costs of $16,335.91 compared with gMTRs, resulting in an incremental cost-effectiveness ratio (ICER) of $26,383.82 per QALY gained (Table 10).

**Table 10:** Cost-effectiveness of STRs versus gMTRs for the treatment of HIV-1 infection in the United States.

	STR	gMTR	Δ STR-gMTR
**Total Costs**	$547,540.20	$531,204.29	$16,335.91
** Medication Costs**	$450,474.20	$423,926.76	$26,547.43
** Inpatient Costs**	$31,258.18	$43,293.79	-$12,035.61
** Other Costs**	$65,807.83	$63,983.73	$1,824.09
**Life Years**	15.400	14.785	0.614
**QALY**	14.466	13.847	0.619
		**ICER** ($/QALY)	**$26,383.82**

gMTR, generic multiple-tablet regimen; ICER, Incremental cost-effectiveness ratio; QALY, Quality adjusted life years; STR, Single-tablet regimen.

Undiscounted life expectancy after ART initiation was estimated to be 22.4 years for STR and 21.1 years, with a benefit of 1.3 years in favor of the STR treatment. The undiscounted QALY gain for STR over gMTR was 1.3 QALY for a marginal lifetime cost of $41,188.85 resulting in an undiscounted ICER of $31,910.87 per QALY gained.

### Sensitivity Analysis

**Fig 5:**
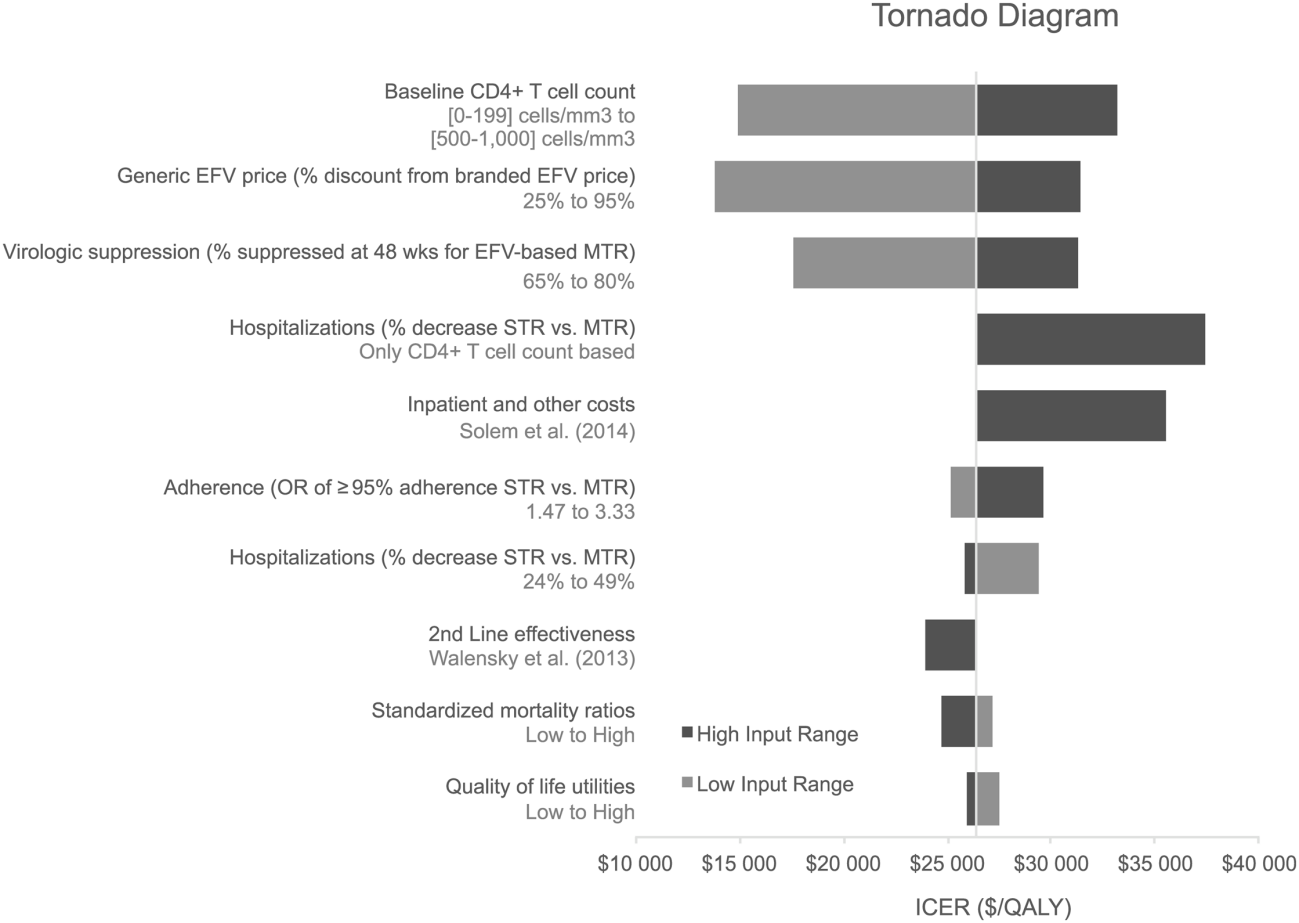
Tornado diagram of univariate analyses showing the degree to which uncertainty in individual variables affects ICER ($/QALY). EFV, efavirenz; ICER, incremental cost-effectiveness ration; MTR, multiple-tablet regimen; QALY, quality adjusted life years; STR, single-tablet regimen.

A deterministic sensitivity analysis was conducted to determine the factors that had the greatest influence on the ICER. The range of variation to each parameter is described in the S1 Table. The discounted lifetime ICER for HIV treatment initiation with STRs compared with gMTRs was $26,384 per QALY in the base case and ranged from $13,790 to $37,438 per QALY (Fig 5).

As shown in the Tornado diagram, the ICER increased when baseline CD4 cell count, gEFV price discount, percentage of patients suppressed at 48 weeks for EFV-based gMTR and odds ratio of ≥95% adherence are assumed higher than in the base case. The opposite is true for a higher percentage decrease in hospitalizations on STR vs. MTR, higher standardized mortality ratios and higher quality of life utilities.

Considering second-line therapy effectiveness equal to that considered in Walensky et al. (2013) [7] decreased the ICER to $23,895 per QALY. Allowing hospitalization costs to be driven exclusively by CD4 counts for both STR and MTR results in an ICER of $37,438 per QALY. Considering a different source of inpatient and other costs [10] similarly increases the ICER to $35,521 per QALY. Finally, assuming, upon failing 6^th^-line therapy, a successive sequence of further therapy lines with efficacy, safety and cost parameterization equal to that of the 6^th^-line, results in a dominant scenario for STR as compared to gMTR: an increase of 0.49 discounted QALY, accompanied by $14,348 in discounted total cost savings.

The cost-effectiveness results were most sensitive to baseline CD4+ T cell count, generic EFV price (% discount from branded EFV price) and 48 weeks virologic suppression rates for EFV-based gMTR. Sensitivity analysis to the values of other variables like the odds ratio of ≥95% adherence between STR and MTR, hospitalizations rates, second line-treatment effectiveness, standardized mortality ratios, quality of life utilities and costs revealed minor uncertainty in the range of 2% to 12% relative to baseline ICER.

**Fig 6:**
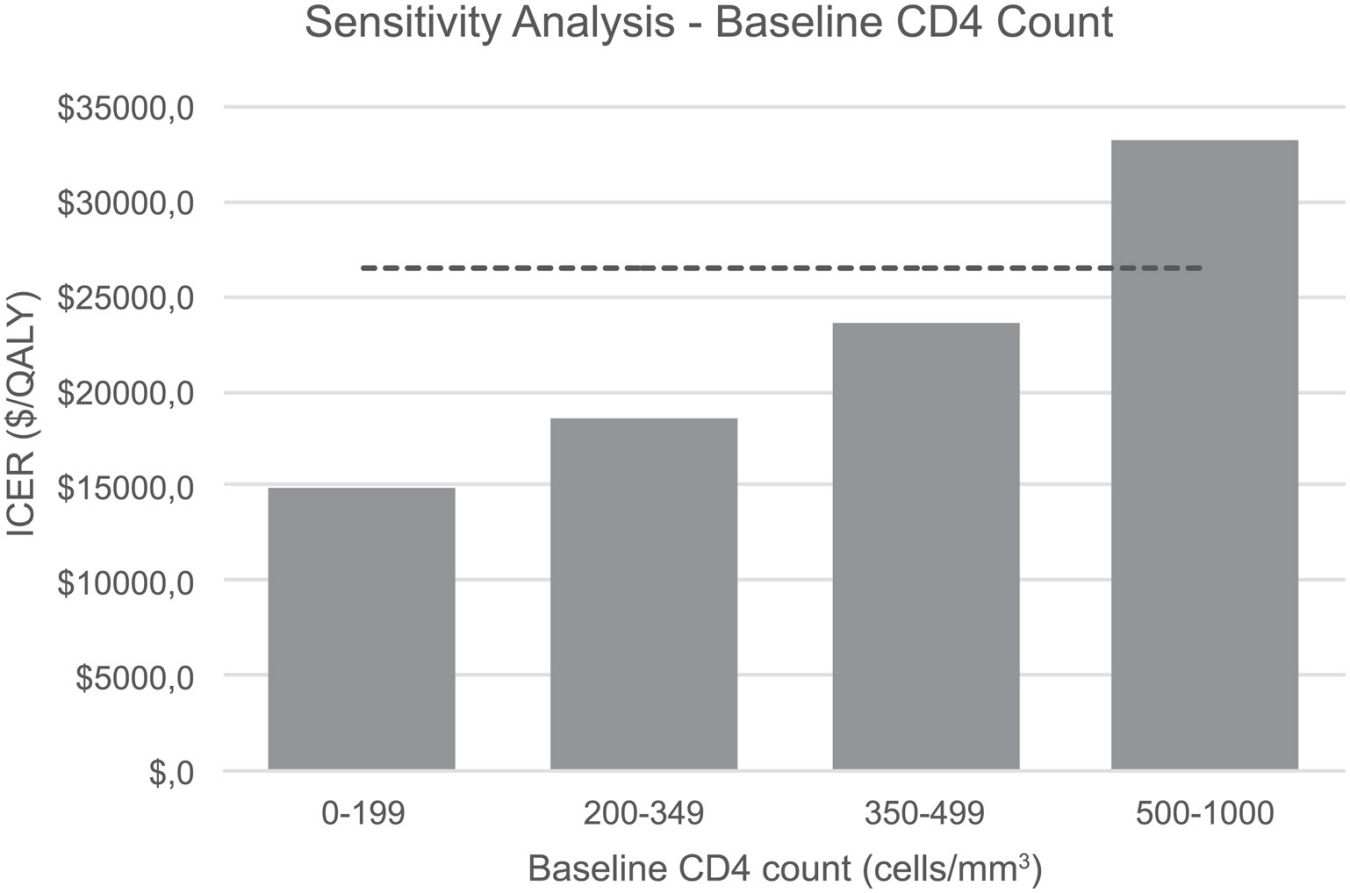
One-way sensitivity analysis for baseline CD4 count by class. ICER, incremental cost-effectiveness ration; QALY, quality adjusted life years.

The assumption about the value of baseline CD4 count had the most impact in the variability of the ICER of STR over gMTR (Fig 6). It ranged between $14,911/QALY and $33,211/QALY in patients with baseline CD4 count of 0–199 cells/mm^3^ to 500–1000 cells/mm^3^, respectively.

**Fig 7:**
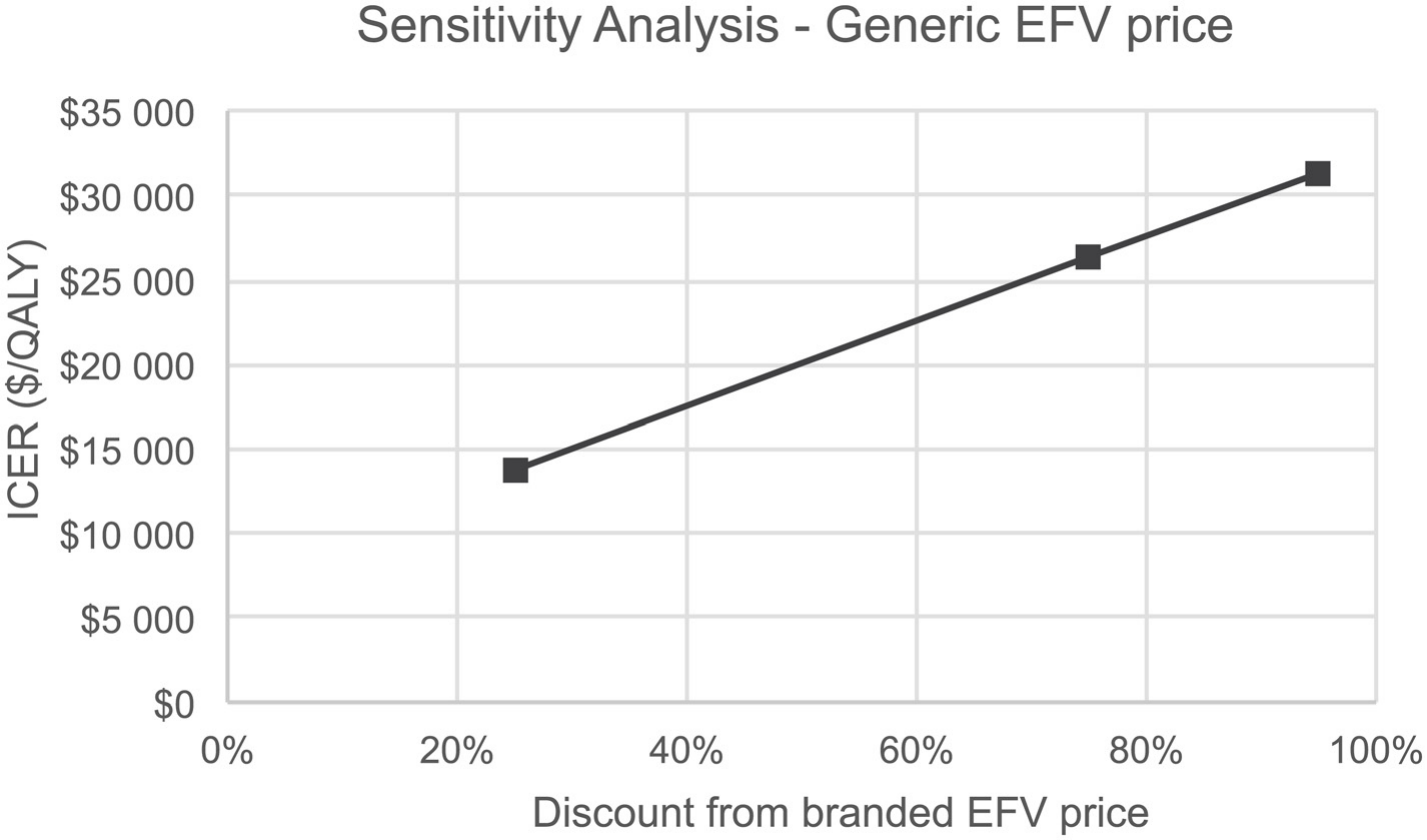
One-way sensitivity analysis for generic efavirenz price reduction. EFV, efavirenz; ICER, incremental cost-effectiveness ration; QALY, quality adjusted life years.

The base case analysis already incorporated a substantial discount of 75% on the price of generic relative to branded efavirenz. Varying the price generic efavirenz up to a 95% discount was associated with a maximum ICER of $31,421/QALY (Fig 7). This value is still well below commonly used cost-effectiveness thresholds in US [45].

## Discussion

In this study we introduce a comprehensive model to assess the lifetime health and economic outcomes of HIV infected patients initiating STRs compared with MTRs including generic medications where possible. STRs considered are those composed of TDF/FTC and efavirenz or rilpivirine or elvitegravir/cobicistat. MTRs considered included the counterparts to STRs substituting emtricitabine by generic lamivudine (g3TC) and branded efavirenz by the generic. The underlying motivation was to understand the value for money of branded STRs in comparison to MTRs composed of generic medications available in the U.S. market.

It is estimated that initial treatment for HIV infection with STRs would result in an enhanced life expectancy of 1.3 years (undiscounted) in comparison to use gMTRs. Lifetime health care costs are estimated to be higher with STR and partially offset by higher inpatients costs expected with gMTR treatment. We estimate a mean increment of $16,336 in lifetime health care costs with STR treatment over gMTR corresponding into an incremental cost of $26,384 per QALY. A sensitivity analysis to the key parameters of the model showed low level of uncertainty with ICER ranging from $13,790 to $33,211 per QALY.

Our estimates of discounted lifetime medical costs of $547,540 in HIV-infected individuals starting up-to-date STRs are comparable with other recently published model based estimates ($591,400) using a similar set of assumptions in patients initiating standard of care [46]. Also, mean life expectancy of 65.4 years and discounted quality adjusted life years of 14.5 QALY after ART initiation at the age of 43 years predicted by our model are within the range of previous research suggesting life expectancy values between 63 and 68.5 years and gains of 12.5 to 16.4 QALY [7, 46, 47]. Nonetheless, substantially lower discounted lifetime treatment costs ranging from $326,500 to $342.800 can be found in other studies, contrasting with our results [7, 47]. These differences may be explained by the non-inclusion of costs other than ART [7] or different assumptions about ART adherence or the risk of mortality modeling leading to a likely underestimation of lifetime medical costs [47].

Our study is subject to some limitations. The gMTR comparator in our study includes generic efavirenz and lamivudine in substitution of branded efavirenz and emtricitabine of EFV/TDF/FTC, RPV/TDF/FTC and EVG/COBI/TDF/FTC single-tablet regimens. In the literature, no direct evidence was found comparing these STRs and an MTR of RPV or EVG/COBI. In this case a simplifying assumption was made assuming the same relative measure of efficacy and safety between gEFV+TDF+g3TC and EFV/TDF/FTC (Study GS-236-102 and Study 903) [18–20, 23, 24], without performing any indirect comparison, nor controlling for any imbalances in study populations or trial protocols and definitions. This assumption was assessed in sensitive analysis by changing the virologic suppression rate for EFV-based MTR, which changes the odds ratios.

Although the gMTR comparators are not recommended options by the DHHS Panel on Antiretroviral Guidelines for Adults and Adolescents [48], as the present analysis aimed at highlighting the losses associated with strategies driven by costs, this means that non-recommended strategies could be selected, as long as they lead to short-term cost-savings. In fact, some authors already reported the utilization of generics as a strategy to reduce costs with ART medication [6]. Other limitation that could be pointed is the non-inclusion of the new STR composed of dolutegravir, abacavir and lamivudine (DTG/ABC/3TC) as a first-line option. This is easily explained by the recent marketing of this STR which was not already in the market at the time of the model implementation. Nevertheless, besides DTG/ABC/3TC still having a low market share, if it was considered in the model it would only improve the results as it is a regimen with only one generic substitute, unlike EFV/TDF/FTC which has two.

Reported efficacy was assumed valid irrespective of the high levels of adherence observed and different measurement techniques used in studies GS-236-102 and 903. In first- and subsequent-line therapies no pooling of efficacy and safety data was performed if different studies were identified reporting on the same ART regimen. Virologic failure probabilities calibrated on our study are relatively low as compared to virologic failure probabilities of other first- and subsequent-line regimens. This can be expected to influence the average time spent in first-line therapy and in consequence, lifetime results.

Because little is known about the adherence-effectiveness relationship in integrase inhibitor (INI) based regimens for modeling purpose this relationship was based on results of populations mainly treated with EFV-based therapy (>60%) and variable NRTI-backbones [37, 49]. Differential adherence between STRs and gMTRs is assumed to influence virologic response for first-line therapy only. It has been suggested, however, that non-adherence to second-line regimens is associated with non-adherence to first-line regimens [50].

The STR associated benefit on adherence over MTR is based on Sweet et al. (2014) [17] and Sax et al. (2012) [4], who compare adherence of STR to adherence of any MTR, including not only generic based equivalent drugs of the STR, but also regimens with other drugs. As such, it was assumed that the observed STR benefit on adherence results from the STR formulation, and not, for instance, from a potentially better tolerability profile of the STR components as compared to the components of the regimens included in the MTR considered in Sweet et al. (2014) [17] and Sax et al. (2012) [4]. As more reliable adherence data become available, these could robustify the current analysis.

Patients discontinuing sixth-line therapy due to virologic failure or other reasons move to a seventh-line, where they are modelled as being in an ART-free non-suppressive state, with HIV-1 RNA viral load levels increasing from their highest value ever observed, and CD4+ T cell counts decreasing from their lowest value ever observed, until death. One might question the assumption used to model this seventh-line, in that it is not clear if patients failing multiple therapy-lines in reality move to an ART-free state. Furthermore, depending on the average time spent in previous therapy-lines and age reached when entering this seventh-line, its influence on overall results might be larger than intended. This was dealt with in a sensitivity analysis, assuming a successive sequence of further therapy lines with efficacy, safety and cost parameterization equal to that of the sixth-line therapy.

Due to the computational expensiveness of the model, traditional probabilistic sensitivity analysis through a large number of model runs at different random input values was deemed unfeasible. Solutions to this problem have been suggested in the literature, a common one of which is through regression meta-modelling [51]. The application of these types of methods was outside of the scope of the current project and will be dealt with in future research.

Consistent with other model estimates [52, 53] and real-world economic analyses [54] we also found that STR is a cost-effective therapeutic option in comparison to gMTR. Following Italian guidelines for the initial HIV treatment, Colombo et al. (2011) found that TDF/FTC/EFV one pill per day was the most cost-effective treatment strategy, compared with the other recommended therapeutic regimens, being also cost-effective (ICER €9,189/QALY) relative to other MTR with lower strength of evidence like TDF + 3TC + EFV [52]. A review conducted by the World Health Organization suggests for the clinical interchangeability of FTC and 3TC, but does not pronounce itself on their interchangeability when comparing single to multiple tablet regimens [55]. It further alerts for the fact that development of M184V/I mutations is associated to a greater extent with the use of a 3TC rather than FTC regimen, though the clinical implications of this difference are difficult to predict [55].

Most notably, real-word evidence suggests cost-savings in HIV-infected patients starting ART with STR in addition to health gains [12, 54, 56]. In a study assessing outcomes and costs of ART as a once-daily STR (n = 1,797) or two or more pills per day (MTR, n = 5,584) of Medicaid patients with an HIV diagnosis from 2005 to 2009 the authors report significantly lower total healthcare costs for STR patients (monthly costs of $2,959 vs $3,544 in MTR, p<0.001) due to lower pharmacy costs, fewer hospitalizations and lower hospital costs, explained by lower pill burden and enhanced adherence with STR [12]. An incremental cost-effectiveness analysis of efavirenz, tenofovir, and emtricitabine as a single-tablet regimen versus a multi-pill regimen, with reference to untreated HIV-infected patients, from the perspective of the Italian National Health Service, identified that a 24% price decrease would be needed for MTR to be comparable with STR in terms of the same ICER relative to no treatment [53].

In contrast to this evidence and to our results is the study by Walensky et al. (2013) estimating that the incremental cost-effectiveness for first-line branded STR in United States is over $100,000/QALY relative to gMTR. The authors suggest that starting or switching to generic based regimens would initially yield annual savings approaching $1 billion for programs that fund HIV treatment in the United States, at the expense of a slightly less effective generic alternative (1 QALY loss per patient). But because non-medication costs and the costs for other medications were not included, and adherence to ART was not explicitly modeled, we anticipate that these results may be subject to a high degree of uncertainty and potentially underestimate the total costs associated with gMTR. In fact, breaking down once-daily fixed-dose ART into their single components and switching emtricitabine by less efficacious lamivudine [57] may lead to lower adherence and virologic suppression [7]. Higher pill burden is associated with both significantly lower adherence rates and worse virological suppression [58]. Poor control of HIV [59], greater pill burden [12] and lower adherence [4] are all associated with higher risk of hospitalization and higher inpatient costs and other non-ART costs that may account up-until 45% of total lifetime costs of current HIV Care [10, 47].

Under these circumstances, until a once-a-day STR generic version is available it is likely that current gMTR possibilities may still be unattractive under contemporary cost-effectiveness thresholds ($100,000/QALY) [45], and may not provide sufficient economic savings to justify the permanent health losses, raising significant legal and ethical questions [60].

It should be noted that at the time of analysis in the U.S. no generic fixed dosed combinations (FDCs) were marketed yet. The current analysis is only applicable to scenarios where switching from an STR to generics means a therapy complication. In countries where generic FDCs are available, differences between generic and non-generic STRs will mainly be due to costs differences between both.

In conclusion, our modeled analysis demonstrates that STRs provided to HIV-1-infected treatment-naive individuals is cost-effective compared with gMTRs. We hope the findings from this study will provide further insight and help guide decisions.

## Supporting Information

S1 Table Low input and high input values of the tornado diagram. (DOCX)
