# Supplementary material for: Cost-Effectiveness of Single- Versus Generic Multiple-Tablet Regimens for Treatment of HIV-1 Infection in the United States
Source: PLoS One. 2016 Jan 25;11(1):e0147821. doi: 10.1371/journal.pone.0147821 (PMC4725959; doi:10.1371/journal.pone.0147821)
Supplement: S1 Table — (DOCX) [file pone.0147821.s001.docx]

# Supporting Information

**S1 Table.** Low input and high input values of the sensitivity analysis presented in the tornado diagram

| **Model component** | **Input range** | **Low value** | **High value** |
| --- | --- | --- | --- |
| Baseline CD4+ T cell count | [0-199] cells/mm^3^ to [500-1000] cells/mm^3^ | $14,911.16 | $33,210.91 |
| Generic EFV price (% discount from branded EFV price) | 25% to 95% (assumption) | $13,790.32 | $31,421.22 |
| Virologic suppression (% suppressed at 48 weeks for EFV-based gMTR) | 65% to 80% (assumption) | $17,590.67 | $31,297.25 |
| Hospitalizations (% decrease STR vs. MTR) | Only CD4+ T cell count based |  | $37,437.95 |
| Inpatient and other costs | Solem et al. (2014) [1] |  | $35,520.87 |
| Adherence (OR of ≥ 95% adherence STR vs. MTR) | 1.47 (Kauf et al. (2012) [2]) to 3.33 (Taneja et al. (2012) [3]) | $25,189.26 | $29,566.03 |
| Hospitalizations (% decrease STR vs. MTR) | 24% (Sax et al. (2012) [4]) to 49% (+25%) | $29,388.30 | $25,811.54 |
| 2^nd^ Line effectiveness | Walensky et al. (2013) [5] |  | $23,894.58 |
| Standardized mortality ratios | Low to High | $27,146.12 | $24,750.35 |
| Quality of life utilities | Low to High | $27,445.87 | $25,949.27 |

Legend: EFV, efavirenz; gMTR, generic multiple-tablet regimen; MTR, multiple-tablet regimen; STR, single-tablet regimen.

**References**

1. Solem CT, Snedecor SJ, Khachatryan A, Nedrow K, Tawadrous M, Chambers R, et al. Cost of treatment in a US commercially insured, HIV-1-infected population. PLoS One. 2014;9(5): e98152.

2. Kauf TL, Davis KL, Earnshaw SR, Davis EA. Spillover adherence effects of fixed-dose combination HIV therapy. Patient preference and adherence. 2012;6: 155-164.

3. Taneja C, Juday T, Gertzog L, Edelsberg J, Correll T, Hebden T, et al. Adherence and persistence with non-nucleoside reverse transcriptase inhibitor-based antiretroviral regimens. Expert Opin Pharmacother. 2012;13(15): 2111-2118.

4. Sax PE, Meyers JL, Mugavero M, Davis KL. Adherence to antiretroviral treatment and correlation with risk of hospitalization among commercially insured HIV patients in the United States. PLoS One. 2012;7(2): e31591.

5. Walensky RP, Sax PE, Nakamura YM, Weinstein MC, Pei PP, Freedberg KA, et al. Economic savings versus health losses: the cost-effectiveness of generic antiretroviral therapy in the United States. Ann Intern Med. 2013;158(2): 84-92.
